# Genomics and biochemistry investigation on the metabolic pathway of milled wood and alkali lignin-derived aromatic metabolites of *Comamonas serinivorans* SP-35

**DOI:** 10.1186/s13068-018-1341-3

**Published:** 2018-12-27

**Authors:** Daochen Zhu, Haibing Si, Peipei Zhang, Alei Geng, Weimin Zhang, Bin Yang, Wei-Jun Qian, Murillo Gabriel, Jianzhong Sun

**Affiliations:** 1 Biofuels Institute, School of the Environment and Safety Engineering, Jiangsu University, Zhenjiang, Jiangsu China; 2 State Key Laboratory of Applied Microbiology Southern China, Guangdong Provincial Key Laboratory of Microbial Culture Collection and Application, Guangdong Open Laboratory of Applied Microbiology, Guangdong Institute of Microbiology, Guangzhou, China; 3 Bioproducts, Sciences and Engineering Laboratory, Department of Biological Systems Engineering, Washington State University, Richland, WA 99354 USA; 4 Biological Sciences Division and Environmental Molecular Sciences Laboratory, Pacific Northwest National Laboratory, Richland, WA 99352 USA

**Keywords:** *Comamonas serinivorans* SP-35, Metabolic pathway, Lignin, Whole genome sequencing, Aromatic metabolites

## Abstract

**Background:**

The efficient depolymerization and utilization of lignin are one of the most important goals for the renewable use of lignocelluloses. The degradation and complete mineralization of lignin by bacteria represent a key step for carbon recycling in land ecosystems as well. However, many aspects of this process remain unclear, for example, the complex network of metabolic pathways involved in the degradation of lignin and the catabolic pathway of intermediate aromatic metabolites. To address these subjects, we characterized the deconstruction and mineralization of lignin with milled wood lignin (MWL, the most representative molecule of lignin in its native state) and alkali lignin (AL), and elucidated metabolic pathways of their intermediate metabolites by a bacterium named *Comamonas serinivorans* SP-35.

**Results:**

The degradation rate of MWL reached 30.9%, and its particle size range was decreased from 6 to 30 µm to 2–4 µm—when cultured with C. serinivorans SP35 over 7 days. FTIR analysis showed that the C–C and C–O–C bonds between the phenyl propane structures of lignin were oxidized and cleaved and the side chain structure was modified. More than twenty intermediate aromatic metabolites were identified in the MWL and AL cultures based on GC–MS analysis. Through genome sequencing and annotation, and from GC–MS analysis, 93 genes encoding 33 enzymes and 5 regulatory factors that may be involved in lignin degradation were identified and more than nine metabolic pathways of lignin and its intermediates were predicted. Of particular note is that the metabolic pathway to form the powerful antioxidant 3,4-dihydroxyphenylglycol is described for the first time in bacteria.

**Conclusion:**

Elucidation of the β-aryl ether cleavage pathway in the strain SP-35 indicates that the β-aryl ether catabolic system is not only present in the family of Sphingomonadaceae, but also other species of bacteria kingdom. These newly elucidated catabolic pathways of lignin in strain SP-35 and the enzymes responsible for them provide exciting biotechnological opportunities for lignin valorization in future.

**Electronic supplementary material:**

The online version of this article (10.1186/s13068-018-1341-3) contains supplementary material, which is available to authorized users.

## Background

Lignin is the most abundant natural aromatic compound on the planet and is a promising source of renewable aromatic building blocks, such as sinapyl alcohol, coniferyl alcohol and *p*-coumaryl alcohol. Because lignin is a highly cross-linked polymer formed by the polymerization of 4-hydroxyphenylpropanoid monomer units such as *p-*hydroxyphenyl (H), guaiacyl (G) and syringyl (S) units and linked by ether or carbon–carbon bonds, its complicated 3-dimensional polymer network structure has a highly recalcitrant nature. However, the biosphere has already developed catabolic pathways for lignin since its introduction into the global carbon cycle [1]. Among the lignin degraders, fungi have been already extensively studied in recent decades; the high oxidative capacity of some fungal enzymes is able to attack lignin and some lignin fragments. The enzymes involved in lignin degradation in fungi include lignin peroxidases (LiP), manganese peroxidases (MnP), versatile peroxidases (VP), dye decolorizing peroxidases (DyPs) and copper-containing oxidative laccases (Lacc). In comparison to fungi, lignin degradation by bacteria has been less studied. However, DyPs have been identified as an important class of oxidases involved in the bacterial degradation of lignin. It has been reported that Lacc and DyPs, were observed as oxidases in lignin degrading bacteria, but without LiP, MnP and VP [2, 3]. Bacteria do not possess the typical peroxidases that fungi employ for lignin degradation, which suggests that the ligninolytic enzymatic system of bacteria might be different from that of fungus.

Recently, an abundant number of species of lignin degrading bacteria have been identified and studied in detail because of their broad growth range and tolerance to extreme environments. For example, *Bacillus ligniniphilus* L1 is a halotolerant and alkaliphilic bacterium which is able to grow in a broad range of temperatures (10–50 °C) and pH (7–11, with the optimal pH 9 for lignin degradation), has great potential for the treatment of high-salt and high-pH wastewater from papermaking, printing, and dyeing [4]. In addition, bacteria were not only able to depolymerize lignin but also played a major role in the mineralization of lignin-derived heterogeneous aromatic compounds [5]. There are multiple metabolic pathways of aromatic compounds that are involved in the mineralization of lignin after lignin depolymerization in bacteria, such as β-aryl ether, biphenyl, protocatechuate, catechol and gentisate pathways. [6, 7]. Dioxygenases play an important role in these pathways, which cleave carbon–carbon bonds of aromatic rings to produce ring-opened products that enter central carbon metabolism via β-ketoadipate pathway (β-KAP) [8]. However, there are still many metabolic pathways that are unknown because hundreds of lignin derivatives have been identified from lignin [9]. Therefore, elucidating these metabolic pathways in bacteria is beneficial for producing high-value aromatic compounds from lignin through genetic manipulations such as knocking out the genes of key step enzymes. So far, most of the literature about the bacterial degradation of lignin used alkali lignin (AL) or kraft lignin as the substrate. However, during the production of AL, sodium hydroxide was used, and the hydrolysis and condensation reactions destroyed the cross-linked structure of lignin, forming different molecule sizes. Compared to AL, milled wood lignin (MWL) is more suitable for the investigation of lignin degradation by bacteria, as it has been typically considered to be the most representative molecule of lignin in its native state [10, 11]. Therefore, it is important to characterize the degradation of lignin by bacteria using MWL as the substrate, examine the intermediate metabolites, and clarify their metabolic pathways during the process of lignin depolymerization and mineralization. The objective of this study was to demonstrate the depolymerization of MWL and AL to produce low-molecular-weight aromatic metabolic intermediates by the strain *Comamonas serinivorans* SP-35 DSM 26136^T^, as well as the reconstruction of the catabolic pathways of those lignin-derived aromatic compounds by gas chromatography–mass spectrometry analysis and whole-genome sequencing and annotation.

## Methods

### Strain and media

*Comamonas serinivorans* SP-35 DSM 26136^T^ was isolated from compost by our lab and identified as a new species of genus *Comamonas* [12]. It was routinely cultured in Luria broth (LB) medium containing (g/L): 10.0 Bactopeptone (Difco Laboratories, Detroit, MI, USA); 5.0 yeast extract (Difco Laboratories); 5.0 NaCl. The lignin incubation medium (LM) used in this study was as follows (g/L): K_2_HPO_4_, 1.0; MgSO_4_, 0.2; CaCl_2_, 0.05; FeSO_4_, 0.05; MnSO_4_, 0.02; KH_2_PO_4_, 1.0; (NH_4_)_2_SO_4_, 2.0; and 0.5 g MWL or AL (CAS 8068-05-1, Catalog number 370959, Sigma-Aldrich, St. Louis. Mo) as carbon source.

### Milled wood lignin preparation

The isolation and purification of MWL were performed as described before [13]. The corncobs (collected from a farm from Yuncheng County, China) were dried at 50 °C for 12 h and milled to a size under 40 meshes. Then, the samples were extracted using ethanol for 4 h and a benzene/ethanol (2:1, v/v) solution for 8 h using a Soxhlet extractor and dried. Then, the samples were ground (600 rpm) for 72 h in a vibratory ball mill with stainless steel balls and toluene. Afterwards, the ball-milled wood samples were extracted twice with dioxane/water (2 × 200 mL; 96:4, v/v) for 24 h under a nitrogen atmosphere, and the supernatant was freeze-dried to obtain the crude MWL. The crude MWL was dissolved in acetic acid/water solution (9:1, v/v), dropped into deionized water overnight, and then centrifuged and freeze-dried. The crude lignin dissolved in dichloroethane/ethanol (2:1, v/v) was added to diethyl ether dropwise, and the un-dissolved fraction was separated by centrifugation and freeze-dried to obtain the purified MWL.

### Growth of strain SP-35 and remove of chemical oxygen demand

Cells of SP-35 were inoculated in LB medium at 30 °C for 18 h, and then the pellets were collected by centrifugation and washed twice with a potassium phosphate buffer (100 mM) to remove the residual medium and re-suspended in 0.9% (v/v) NaCl solution. For growth culture, 1 mL of re-suspended cells were inoculated in 100 mL of LM medium with 0.05 g of MWL or glucose as the carbon source or without carbon source as control and incubated in the shaker at 30 °C, 220 rpm. For the measurement of chemical oxygen demand (COD), supernatant was collected by the centrifugation of culture at 10,000 rpm for 5 min. The dichromate method (standard methods, Section 5220D, Eaton et al. [14]) was used to determine COD [14]. All measurements were conducted in triplicate.

### Scanning electron microscopy analysis

For scanning electron microscopy (SEM) analyses, the samples were centrifuged and the supernatants were freeze-dried. Afterwards, the samples were mounted on aluminum stubs, coated with a gold–palladium alloy, and examined with scanning electron microscopy (SEM, JSM-7001F, Japan).

### Fourier-transform infrared spectrometer spectra analysis

Fourier-transform infrared spectrometer (FTIR) spectra analysis was performed with a Bruker Tensor 27 spectrometer (Bruker Optics, Bullerica, MA), 0.001 g ground sample was mixed with 0.1 g KBr and analyzed in the range of 4000–400 cm^−1^. The absorbance between 250 and 400 nm was measured.

### Gas chromatography–mass spectrometry analysis

For gas chromatography-mass spectrometry (GC–MS) analysis, samples were prepared as described previously [4]. For the silylation procedure, 100 μL dioxane and 10 μL pyridine were added into samples and vortexed in glass tubes followed by silylation, which was performed with 50 μL of trimethylsilyl [BSTFA (N, *O-*bis (trimethylsilyl) trifluoroacetamide) and TMCS (trimethylchlorosilane)], Sigma-Aldrich, (Sigma-Aldrich, St. Louis. MO). The mixture was heated in a water bath at 80 °C for 45 min with periodic shaking to dissolve the residues. GC–MS (AntoSystem XL GC-TurboMass; Perkin-Elmer, Waltham, MA, USA) analysis was carried out with PE-5MS capillary column (20 m × 0.18 mm internal diameter, 0.18 mm film thickness). The injection volume was 1 μL at a carrier gas flow of 1 mL min^−1^ of helium. The column temperature program was 50 °C (5 min); 50–300 °C (10 °C min^−1^, holding time: 5 min). The transfer line and the ion source temperatures were maintained at 200 and 250 °C. A solvent delay of 3.0 min was selected. In the full-scan mode, electron ionization mass spectra in the range of 30–550 (m/z) were recorded at an electron energy of 70 eV. All standard monomeric phenolic compounds (1 mg) were derivatized and chromatograph analyzed as above. To identify the low molecular weight lignin-related compounds as trimethylsilyl (TMS) derivatives derived from bacterial treatment, their mass spectra were compared with that of the data of the GC–MS spectral library (Wiley, NIST) available in the instrument and by comparing the retention time with those of some authentic compounds available.

### Genome sequencing and analysis of *Comamonas serinivorans* SP-35

The genomic DNA of strain SP-35 was extracted using a Wizard Genomic DNA Purification Kit (Promega, Madison, WI) and whole-genome sequencing was performed by the Majorbio Company (Shanghai) using the PacBio RS II platform and done using the single molecule real time (SMRT) sequencing. A SMRT bell sequencing library with a 7–8 kb insert size was prepared using the PacBio SMRTbell Template Prep Kit 1.0. Afterwards, the raw data was assembled with the assistance of a Celera assembler and polished by Quiver tool (version 1.1.0) [15].

## Results and discussion

### Growth of SP-35 and removal of COD

**Fig. 1:**
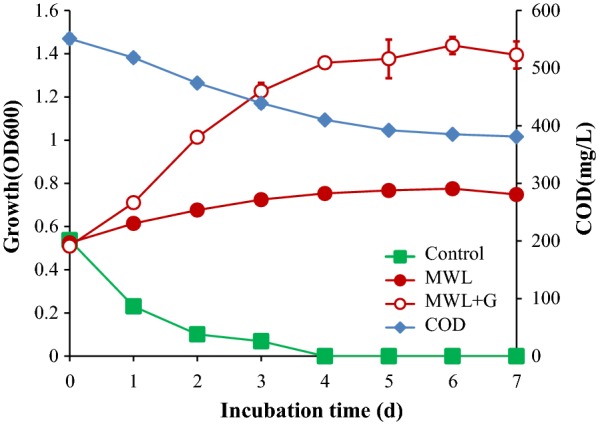
Growth of the strain SP-35 during 7 days of incubation with MWL and glucose as carbon source. Symbols: closed circles, MWL as single carbon source; open circles, MWL and glucose as carbon source; closed squares, control, the growth without carbon source; closed diamond, COD with lignin as carbon source

The strain SP-35 was able to slightly grow (OD_600_ from 0.52 increased to 0.78) with MWL as the sole carbon source and had a faster proliferation when glucose was added as mixed carbon source; cells quickly entered into the decline phase without supplies of carbon sources (Fig. 1). These results indicate that the strain SP-35 is able to utilize MWL as the sole carbon source for cell growth to a limited extent. Moreover, strain SP-35 also utilized AL as a carbon source as described in previous studies [16].

The degradation rate of lignin by strain SP-35 can be determined by COD removal analysis because MWL contributes almost the entire COD loading in the culture medium when it was the sole carbon source. Our results showed that the COD of the strain SP-35 culture was reduced from 551 to 381 mg/L after 7 days of incubation with MWL as the carbon source (Fig. 1). It was further confirmed that the MWL was degraded by the strain SP-35, yielding a 30.9% reduction in lignin content. It is worthy to mention that previously published data reported the reduction of AL by strain SP-35 was 44.4% [16]. It is still unclear why AL is more susceptible to degradation by strain SP-35 than MWL. It should be pointed out that since the structure of MWL is closer to that of native lignin, the degradation rate of strain SP-35 to MWL represents the ability of the strain to degrade natural lignin.

**Fig. 2:**
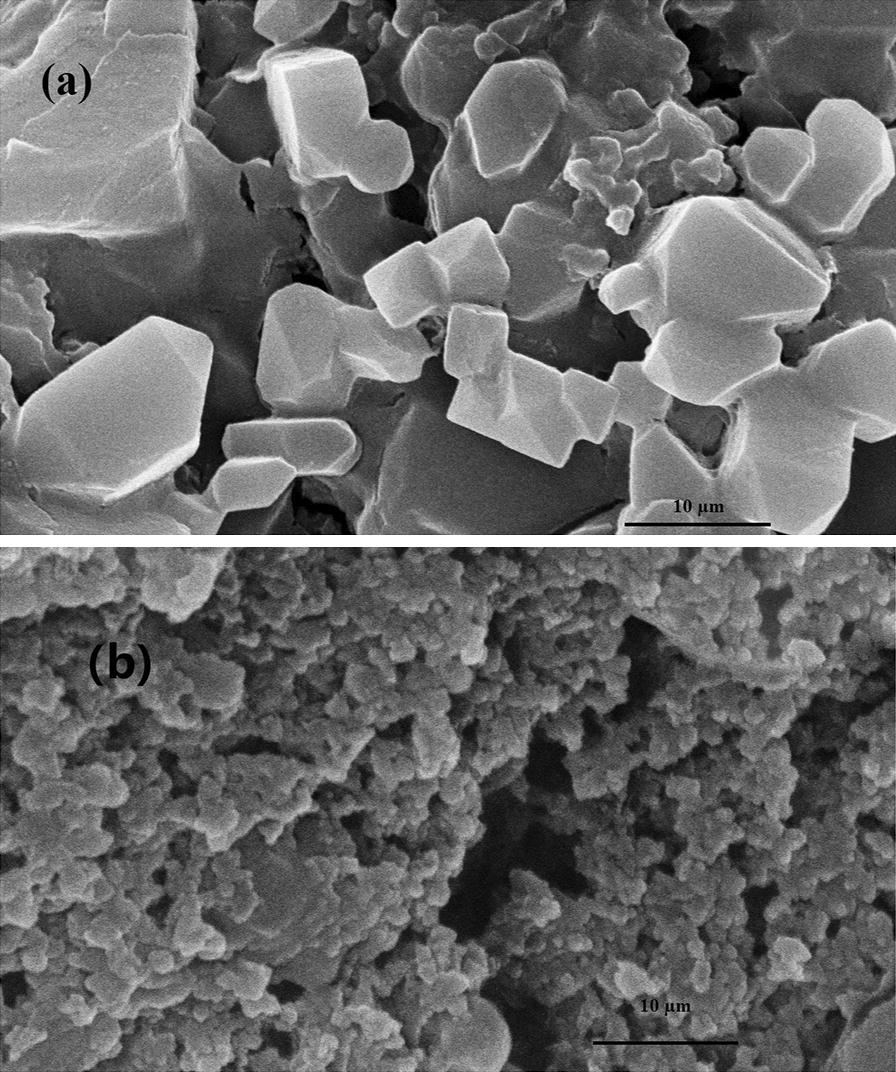
Scanning electron micrograph of lignin. **a** MWL in un-inoculated LM medium incubated for 7 days; **b** MWL treated by 7 days of incubation LM medium with the strain SP-35

To further investigate the bacterial degradation of MWL, both the un-inoculated and inoculated MWL with strain SP-35 were incubated for 7 days and were observed with SEM (Fig. 2). The size of the MWL particles treated by strain SP-35 was significantly smaller than that of the un-inoculated control samples and was reduced from 6–30 µm to 2–4 µm. in addition, the similar phenomenon that lignin particles become smaller also appears in other lignin degradation bacteria [4]. Meanwhile, the morphology of MWL changed from an irregular polyhedron shape to a ball shape. The variation in size and shape of the MWL particles suggest that MWL was degraded and modified.

### Fourier-transform infrared spectrometer spectra analysis

**Fig. 3:**
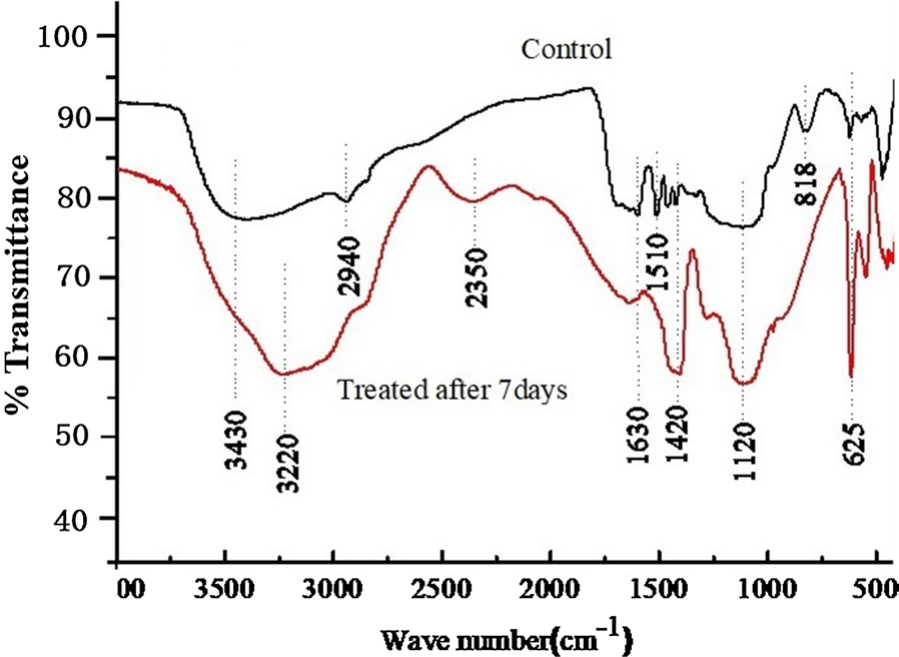
FT-IR infrared spectra of the MWL fractions degraded by the strain SP-35 in LM medium after 7 days incubation

The variability in the chemical composition of lignin can be identified using spectrophotometric methods because the variation in the absorbance of the individual phenolic constituents reflects the change in the chemical structures of the lignin samples. The infrared spectra of the MWL fractions that were degraded by SP-35 for 7 days and the control are illustrated in Fig. 3. The control MWL sample showed a broad band at 3410–3460 cm^−1^, which originated from the hydroxyl groups in phenolic and aliphatic structures, and at 2938–2942 cm^−1^, which arose from C–H stretching in aromatic methoxyl groups and in the methyl and methoxylene groups of the side chains. There are two absorption peaks around 1500 and 1630 cm^−1^, respectively, which originated from the skeletal and stretching vibrations of benzene rings [17]. The C-H bending vibration in methyl groups can be assigned to the band of 1420 cm^−1^ and the absorbance peak at 2350 cm^−1^ ascribed to C=O vibration in ketone groups. Interestingly, compared with the control sample, in the infrared spectra of MWL treated with strain SP-35 the bands from 2938 to 2942 cm^−1^ were weakened. FTIR results suggest that the part of C–C and C–O–C bonds between the phenyl propane structural units of lignin may be oxidized and broken, which result in the reduced vibration of C-H bonds and in the modification of side chains such as OH groups. Another interesting finding was that the absorption peak at 1630 cm^−1^ was weakened and the displacement of the absorption peak at 1120 cm^−1^ was enhanced probably due to C–O stretch, which might be due to the production of new phenyl aromatic compounds as well as small molecules of alcohols or ethers during the process of the degradation of MWL. The appearance of carbonyl group in the range of 1650–1710^−1^ represents C=O bond is in conjugation with the aromatic ring, the variation suggests the production of aldehydes and acids. In addition, the vibration of the absorption peak at 818 cm^−1^ was weakened, proving that the side chain structure of MWL was modified. The FT-IR spectra indicated that the degradation of MWL by strain SP-35 included the oxidative cleavage of the lignin aromatic ring structures and the modification of their side chains.

### Aromatic intermediates identified by gas chromatography–mass spectrometry analysis

**Fig. 4:**
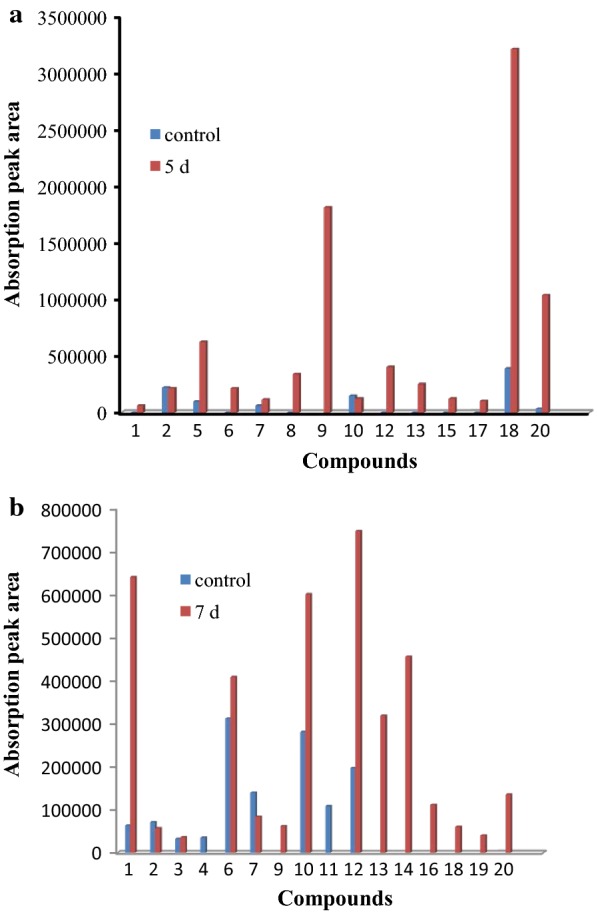
Comparison of peaks of aromatic compounds identified by GC–MS in the first and fifth days of incubation of MWL (**a**) and at first and seventh days of incubation of AL (**b**). Numbers representing the aromatic compounds are shown in Table 1

**Fig. 5:**
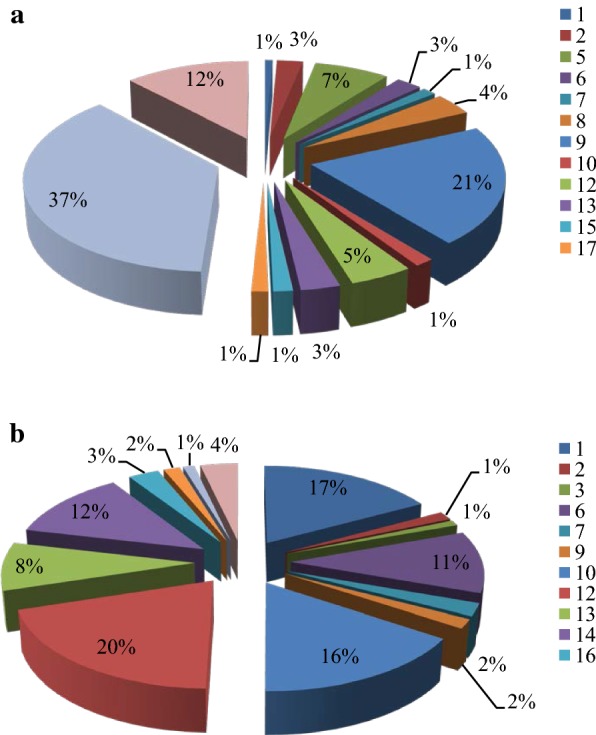
Proportion of peaks areas of aromatic compounds in the fifth day of incubation of MWL (**a**) and seventh day of incubation of AL (**b**). Numbers representing the aromatic compounds are shown in Table 1

To determine the intermediate metabolites, cells of SP-35 were incubated in LM medium with MWL or AL as the sole carbon source for 7 days and the fractions were collected and detected with GC–MS from cultures in the first, third, fifth and seventh day. Hence, a total of 20 single-ring aromatic compounds were identified in both the MWL and AL lignin cultures (Table 1). As can be seen from parts A and B of Fig. 4, most of the absorption peak areas of the aromatic compounds identified from lignin cultures increased during the 7-day incubation and reached its highest point during the 5th and 7th days in the MWL and AL cultures, respectively. Notably, the peak area of certain aromatic compounds significantly increased compared with the control, such as that of ferulic acid (compound 20), which increased 29.3 times, the peak of 4-hydroxycinnamic acid (HCA, compound 18), which increased 8.3 times, and the peak of 2,4-dihydroxybenzaldehyde (2,4-DHB, compound 5), which increased 6.4 times in MWL cultures (Fig. 4a). In addition, the peak area of guaiacol (compound 1), 4′-hydroxyacetophenone (HAP, compound 12), vanillin (compound 10) and 3,4-dihydroxyphenylglycol (DHPG, compound 6) increased 10.2, 3.8, 2.1 and 1.3 times in AL cultures, respectively (Fig. 4b). Therefore, the increased proportion in the peaks of aromatic compounds in the fifth day of incubation of MWL indicated that HCA was the most abundant aromatic metabolite and constituted 37% of aromatic metabolites. There were other major aromatic compounds, including 4-hydroxybenzoic acid (HBA, compound 9, 21%), ferulic acid (12%), 2,4-DHB (7%), and HAP (5%); these aromatic compounds, together with HCA, accounted for 82% of all aromatic metabolites (Fig. 5a). Unlike in MWL, the predominant aromatic compounds in AL culture included HAP (20%), guaiacol (17%), vanillin (16%), 3,4-dihydroxymandelic acid (DOMA, compound 14, 12%), DHPG (11%), and vanillic acid (compound 13, 8%), which together constituted 83% of all aromatic metabolites (Fig. 5b). However, previous studies have shown that the major degradation products of AL when degraded by the strain *Bacillus ligniniphilus* L1 included vanillic acid (44.2%), HAP (14.5%), vanillin (8.7%), and 4-hydroxyphenylacetic acid (7.2%) [4]. Based on these results, we suspected that strain SP-35 and strain L1 have different metabolic pathways for lignin degradation and the production of their respective intermediates. To further explore this claim, metabolic pathway analysis based on whole-genome sequencing should be performed. It must be pointed out that the uncultured MWL also contained small amounts of monomers such as 3-methylphenol (3-MP, compound 2), 2,4-DHB, butylated hydroxytoluene (BHT, compound 7), vanillin, HCA and ferulic acid. The reason why uncultured MWL also contained small amounts of monomers, probably is due to the cleavage of β-*O*-4 linkages and ester bonds (acetyl and coumaryl residues) caused by condensation and oxidation of side chains during the preparation of MWL., Although the single ring compounds are produced during the preparation of MWL, but wouldn’t significantly change the core of the lignin structure [18]. In addition, the tolerance of strain SP-35 to the aromatic compounds was tested with more than 20 aromatic compounds, the results indicate that when the concentration of the compound was 0.1%, strain SP-35 was able to grow under the presence of most of the tested compounds, respectively. When the compound concentration was increased to 0.5%, the growth of cells was observed only with 14 compounds as substrates, and when the compound concentration was increased to 5%, the cells could not grow in the presence of any compound (Additional file 1). Our findings suggest that strain SP-35 could be used as a potential cell factory for the production of high-value aromatic compounds with lignin that might be widely used in the pharmaceutical, fine chemicals, and food industries.

**Table 1:** Aromatic metabolites of lignin degradation by strain SP-35 lignin

No.	Retention time		MWL	MWLC	AL	ALC
1	13.644	Guaiacol	+	–	+	–
2	14.208	3-Methylphenol	+	+	+	+
3	14.82	Phenylacetic acid	–	–	+	+
4	16.192	4-Hydroxybenzaldehyde	–	–	–	+
5	16.23	2,4-Dihydroxybenzaldehyde	+	+	–	–
6	16.286	3,4-Dihydroxyphenylglycol	+	–	+	+
7	17.622	Butylated hydroxytoluene	+	+	+	+
8	17.979	Methyl 2,5-dihydroxybenzoate	+	–	–	–
9	18.496	4-Hydroxybenzoate	+	–	+	–
10	18.562	Vanillin	+	+	+	+
11	18.872	Acetovanillone	–	–	–	+
12	19.549	4′-Hydroxyacetophenone	+	–	+	+
13	20.339	Vanillic acid	+	–	+	–
14	20.678	3,4-Dihydroxymandelic acid	–	–	+	–
15	20.734	Syringaldehyde	+	–	–	–
16	21.797	4-(3-hydroxybutyl)-2-methoxyphenol	–	–	+	–
17	21.806	Diydroferulic acid	+	–	–	–
18	22.173	4-Hydroxycinnamic acid	+	+	+	–
19	23.452	Butyl 2-ethylhexyl phthalate	–	–	+	–
20	23.856	Ferulic acid	+	+	+	–

*MWL* milled wood lignin, *MWLC* control of MWL, *AL* akaline lignin, *ALC* Control of AL

### Lignin degradation and its intermediates metabolic pathways analysis based on the whole-genome sequencing and annotation

With the aim of getting a better understanding of the mechanisms of lignin degradation by strain SP-35 and exploring the catabolic pathways of intermediate metabolites, the whole genome sequence of strain SP35 was obtained by the SMRT method, using the PacBio RS II platform (Whole Genome Shotgun project has been deposited at DDBJ/EMBL/GenBank under the accession no. CP021455.1). Based on the annotation analysis (Additional file 2) of the genome of the strain SP-35, a total of 94 genes that may be involved in the degradation and metabolism of lignin were identified (Table 2). These genes belong to 34 enzymes, one transcriptional regulator, one degradation regulator and three transporters, respectively. It is notable that the genome of the strain SP-35 contained a DyPs and one Lacc encoding genes that is probably involved in the oxidation of lignin, but no LiP or MnP genes were observed. It is supported by published data which showed that the maximum crude enzyme activity of DyPs reached 648.4 u/L while Lacc only reached 70.1 u/L in the fourth day of the culturing of strain SP-35 [16]. The gene sequence identities of encoding DyPs of strain SP-35 with that of *Pseudomonas* (CP015878.1) and *Rhodococcus* (FN563149.1) are 73–69%, however, there are no significant similarity found with that of fungi (Polyporaceae sp. U77073.1). According to the sequence information of Genbank, the fungal and bacterial DyPs show hardly sequence homology and belong to different protein families and subfamilies [19, 20]. In addition, there were four alkylhydroperoxidase, one glutathione peroxidase, and three superoxide dismutase encoding genes observed in the genome of strain SP-35, which might also be involved in the degradation of lignin. Therefore, the DyPs of strain SP-35 may have a potentially great application in lignin degradation, dye discoloration, wastewater treatment, etc.

**Table 2:** Putative enzyme’s genes of strain SP-35 involving in lignin degradation and catabolic

No	Encode protein	Orf-name	KO/Gene_ID
1	Dyp-type peroxidase	orf02723_1	K07223
2	Alkylhydroperoxidase	orf01844_1, orf01464_1, orf00109_1, orf01615_1	
3	Glutathione peroxidase	orf01521_1	K00432
4	Laccase	orf02918_1	K05810
5	Cytochrome P450	orf00617_1, orf01862_1, orf00094_1	
6	Superoxide dismutase	orf00200_1, orf01398_1, orf02451_1	K04565, K04564
7	Benzoate 1,2-dioxygenase	orf01595_1, orf01596_1	K05549, K05550
8	1,6-Dihydroxycyclohexa-2,4-diene-1-carboxylate dehydrogenase	orf01598_1	K05783
9	Catechol 1,2-dioxygenase (CatA)	orf01594_1	K03381
10	Muconate cycloisomerase (CatB)	orf01593_1	K01856
12	2,4′-Dihydroxyacetophenone dioxygenase	orf00887_1	
13	4-Hydroxybenzoate polyprenyltransferase	orf04164_1	K03179
14	Phenylacetate-CoA ligase (paaK)	orf02154_1	K01912
15	1,2-Epoxyphenylacetyl-CoA isomerase (paaG)	orf00429_1, orf01010_1, orf00301_1, orf01175_1, orf03337_1	K15866
16	3-Oxoadipyl-CoA thiolase (paaJ)	orf01279_1	
17	3-Hydroxyadipyl-CoA dehydrogenase (paaH)	orf01817_1	
18	4-Coumarate–CoA ligase	orf00090_1, orf00074_1, orf04303_1, orf00300_1	K00666
19	*p*-Hydroxyphenylacetate 3-hydroxylase	orf00203_1	
20	R)-Benzylsuccinyl-CoA dehydrogenase	orf03060_1	K00249
21	Hydroxycinnamoyl-CoA hydratase-lyase	orf03793_1	K15866
22	Vanillate *O*-demethylase oxidoreductase	orf00093_1, orf01861_1	
23	4-Hydroxybenzoyl-CoA thioesterase	orf02427_1, orf03445_1	K07107
24	Aldehyde dehydrogenase	orf01894_1, orf02026_1, orf01760_1, orf00092_1	K00128
25	Alcohol dehydrogenase	orf02362_1, orf01315_1, orf00315_1	
26	*p-*Hydroxybenzoate hydroxylase/Baeyer–Villiger monooxygenase	orf01314_1	
27	Muconolactone d-isomerase (CatC)	orf01601_1	K03464
28	3-Oxoadipate CoA-transferase subunit A (pcaI)	orf01277_1	K01031
29	3-Oxoadipate CoA-transferase subunit B (pcaJ)	orf01278_1	K01032
30	Carboxymuconolactone decarboxylase (pcaC)	orf00668_1, orf02483_1	K01607
31	Beta-ketoadipate enol-lactone hydrolase (pcaD)	orf01600_1, orf01280_1	K01055
32	Beta-ketoadipyl CoA thiolase (pcaF)	orf02814_1, orf01237_1, orf01537_1, orf02199_1	
33	Glutathione S-transferase	orf00149_1, orf03152_1, orf01651_1, orf01341_1, orf03018_1, orf01342_1, orf02126_1, orf02328_1, orf01306_1, orf00150_1, orf00388_1, orf02845_1, orf00142_1, orf03308_1,orf03594_1, orf01202_1	
34	NAD-dependent alcohol dehydrogenase	orf01643_1, orf03396_1	
35	Beta-ketoadipate pathway transcription regulator	orf01276_1, orf00869_1	
36	Gentisate transporter	orf03503_1	K05548
37	Benzoate transporter	orf00892_1, orf01603_1, orf00360_1, orf03205_1, orf01599_1	K05782
38	Alpha-ketoglutarate transporter (benE)	orf01819_1	K03761
39	Benzoate anaerobic degradation regulator	orf04233_1	

**Fig. 6:**
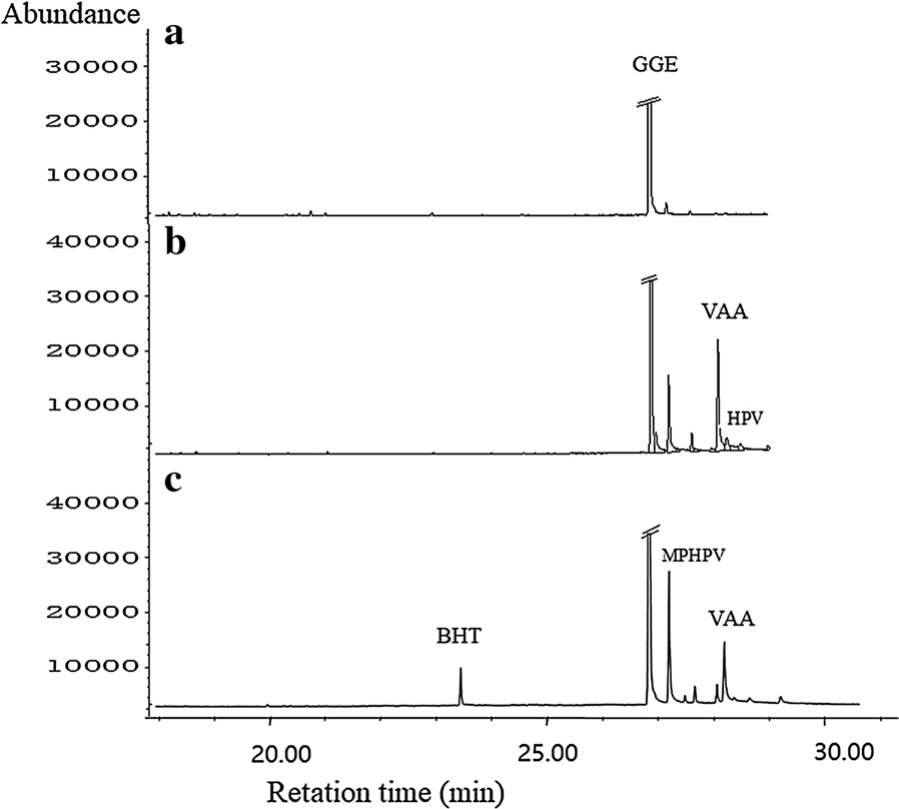
GC-MS spectra of culture of strain SP-35 with GGE as single carbon source. **a** Un-inoculated culture (control); **b** the third day of incubation; **c** the fifth day of incubation. *GGE* guaiacylglycerol-β-guaiacyl ether; MPHPV: α-(2-methoxyphenoxy)-β-hydroxypropiovanillone, *GS-HPV* β-glutathionyl-γ-hydroxypropiovanillone, *HPV* γ-hydroxypropiovanillone, *VVA*: vanilloyl acetic acid

**Fig. 7:**
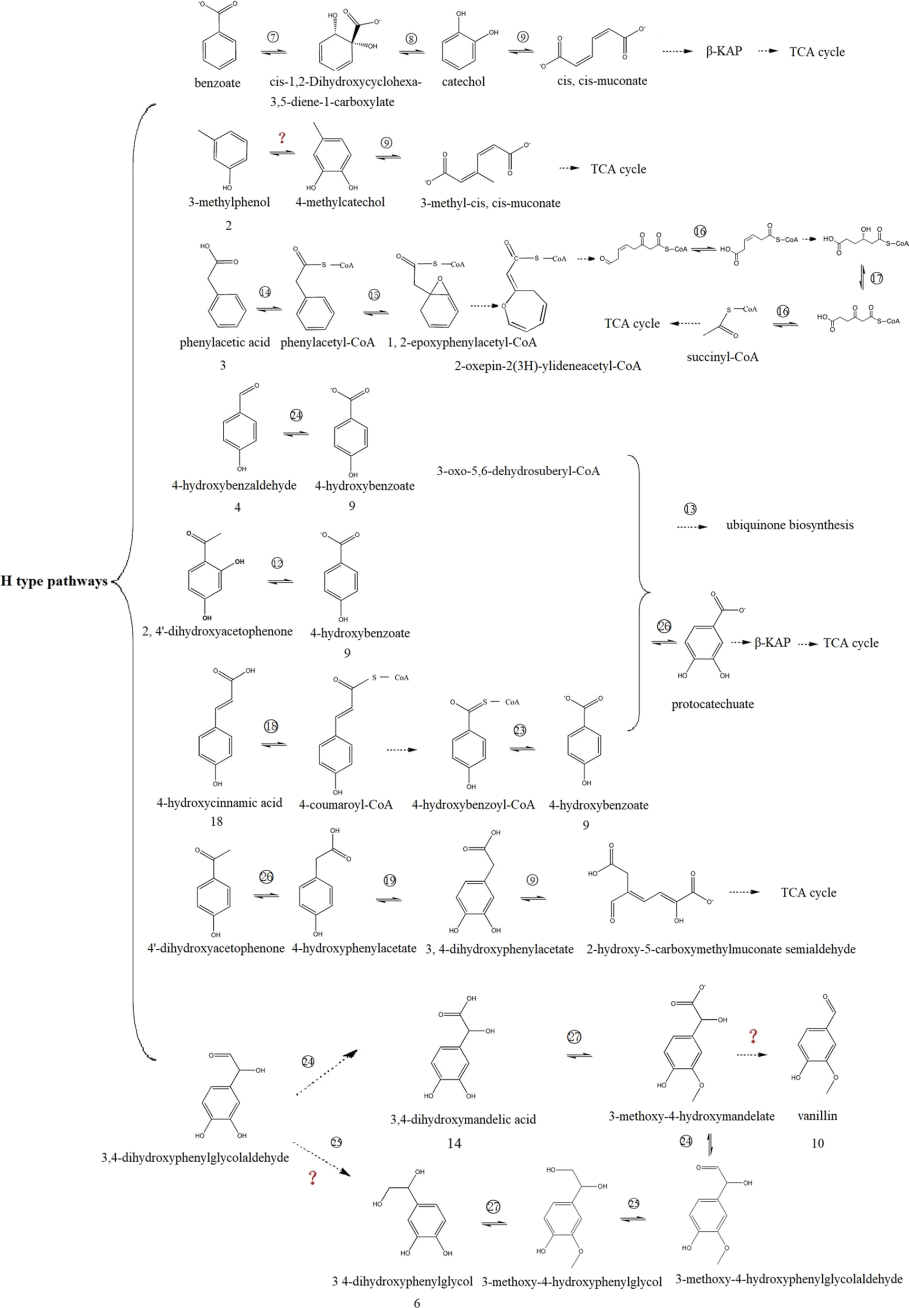
Putative catabolic pathway of lignin derived monomers and catabolism genes in *Comamonas serinivorans* SP-35. H type pathway. Symbol “?” means the enzyme encoding gene was un-confirmed. The reactions indicated by dashed arrows have not been confirmed. Abbreviations: Numbers represent the aromatic compounds were shown in Table 1, and the circled numbers representing the number of enzymes are shown in Table 2

**Fig. 8:**
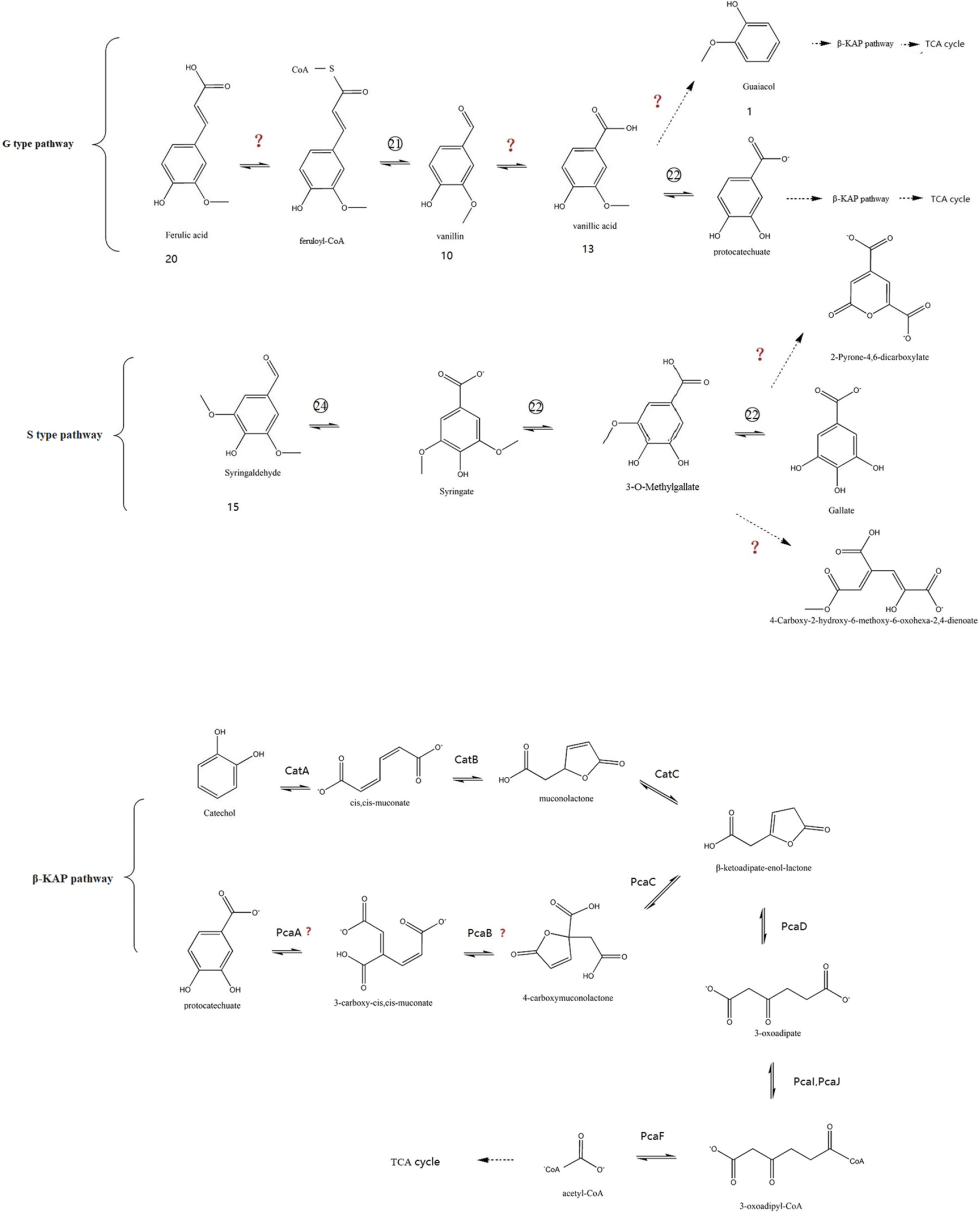
Putative catabolic pathway of lignin derived monomers and catabolism genes in *Comamonas serinivorans* SP-35. G and S pathway and β-KAP. Symbol “?” means the enzyme encoding gene was un-confirmed. The reactions indicated by dashed arrows have not been confirmed. Abbreviations: Numbers represent the aromatic compounds were shown in Table 1, and the circled numbers representing the number of enzymes are shown in Table 2

The β-aryl ether linkage is the most abundant linkage in lignin. It comprises 45–50% of all the intermonomer linkages in softwood and 60–62% of all the intermonomer linkages in hardwood lignin [9]. Genome annotation revealed the presence of sixteen genes of glutathione transferases (GSTs) and two genes of NAD-dependent alcohol dehydrogenase (NAD-ADH) in the genome of strain SP-35, which may be involved in the cleavage of the β-aryl ether linkage, other than that, these genes are also very important for normal celluar process (Table 2). The β-aryl ether cleavage pathway was uncovered in strain *Spihingobuim* sp. SYK-6 by the degradation of guaiacylglycerol-β-guaiacyl ether (GGE). [6]. We speculate that in strain SP-35, two NAD-ADH molecules are probably involved in the conversion of GGE to α-(2-methoxyphenoxy)-β-hydroxypropiovanillone (MPHPV), some GSTs genes with β-etherase activity may participate in the degradation of MPHPV to generate β-glutathionyl-γ-hydroxypropiovanillone (GS-HPV), and some GSTs genes with the activity of GSH-dependent lyase are able to catalyze GS-HPV to produce glutathione disulfide (GSSG) and γ-hydroxypropiovanillone (HPV). The specific substrates and activities of these enzymes and whether they participate in the β-aryl ether cleavage pathway need further validation in the next step work of our investigation. In addition, MPHPV, HPV and vanilloyl acetic acid (VVA) were identified in the third and fifth days of strain SP-35 culture (MM63 medium) with 100 mg/L GGE as solo carbon source incubated 7 days at 30 °C (Fig. 6). In the β-aryl ether cleavage pathway of SYK-6, VVA was obtained from the conversion of HPV [7]. These data further confirmed the presence of the β-aryl ether cleavage pathway in strain SP-35. So far, there are few information available about the β-aryl ether catabolic system except for the three strains of Sphingomonadaceae, and it is believed that the β-aryl ether catalyzed by GSTs as a lignin degradation strategy may exist only in Sphingomonadaceae [7]. Based on the comparison of the homology of the enzyme-encoding gene, the previous articles concluded that the β-aryl ether metabolic pathway may exist only in the Sphingomonadaceae family. However, our results indicate that other species in bacteria kingdom may also have a β-aryl ether cleavage pathway, but the homology of the coding gene of enzymes involving the pathway compare to Sphingomonas is too low to be confirmed based on homology comparison. Thus, the elucidation of the beta-aryl ether catabolic system of strain SP-35 will update the understanding of the role of the beta-aryl ether cleavage pathway in lignin-degrading bacteria. Based on the genome annotations and GC–MS data, we can predict the metabolic pathway of the aromatic intermediate metabolites of strain SP-35 (Fig. 7 and 8). According to the unit type of lignin, the pathways were divided into three categories, including H, G and S types. Among the three categories, the H type has multiple pathways, including benzoate, 3-MP, phenylacetic acid (PA, compound 3), 4-hydroxybenzaldehyde (HBAld, compound 4), HAP, HCA, HAP and DHPG degradation pathways, and both the G and S types have only one pathway, namely the ferulic acid degradation and syringaldehyde (SA, compound 15) degradation pathways, respectively.

The β-KAP is a chromosomally encoded convergent pathway for the degradation of aromatic compounds derived from plants, including lignin, as the primary source of carbon and energy and is widely distributed in soil bacteria and fungi [8, 21]. As we all know, the β-KAP includes two branches of catechol and protocatechuate, and both branches observed should be present in strain SP-35 (Fig. 8). All the enzyme encoding genes involved in the catechol branch have been identified in the genome of strain SP-35, including catechol 1, 2-dioxygenase (*catA*), muconate cycloisomerase (*catB*), muconolactone D-isomerase (*catC*), β-ketoadipate enol-lactone hydrolase (*pcaD*), 3-oxoadipate CoA-transferase subunit A (*pcaI*), 3-oxoadipate CoA-transferase subunit B (*pcaJ*), and β-ketoadipyl CoA thiolase (*pcaF*). In addition, the genes corresponding to the enzymes involved in the PA branch, such as the carboxymuconolactone decarboxylase (*pcaC*) gene, have been observed, but no genes encoding the protocatechuate 3, 4-dioxygenase (*pcaHG*) and β-carboxymuconate cycloisomerase (*pcaB*) were identified. However, protocatechuate was degraded and was used as the sole carbon source by strain SP-35, proving the presence of the protocatechuate degradation pathway (Table 3). It may be due to the homology of *pcaHG* and *pcaB* in strain SP-35 compared with other bacteria was too low to be identified based on sequence alignment, and this hypothesis needs further study to confirm. In addition, a β-KAP transcription regulator (*pcaR*) encoding gene was identified in the genome of strain SP-35 and was required for both the induction of the pca regulon (*pcaBDC*, *pcaIJ*, and *pcaF*) and the chemotactic response of the bacteria to aromatic compounds [22]. It provided additional evidence for the presence of the protocatechuate branch in strain SP-35. In addition, the protocatechuate branch of the KAP pathway of SP-35 differs from the fungus in that 4-carboxymuconolactone is converted to beta-carboxymuconolactone by *pcaB* and then further converted to 3-oxoadipate instead of being converted to beta-ketoadipate-enol-lactone via *pcaC* [8].

**Table 3:** The degradation of aromatic monomers by the strain SP-35

Compounds	Completely degraded time (hours)
Ferulic acid	48
4-Hydroxybenzoic acid	24
4-Coumaric acid	No
Gentisate	24
Protocatechuate	24
Benzoate	168
Vanillin	72
Vanillic acid	36

Benzoate has been widely used as a model compound to study the catabolism of aromatic compounds in bacteria [23]. In strain SP-35, the catabolic pathway of benzoate is as follows: First, benzoate is converted to cis-1, 2-dihydroxycyclohexa-3, 5-diene-1-carboxylate (DHCDC) by the catalysis of benzoate 1, 2-dioxygenase, and is then converted to catechol by DHCDC dehydrogenase. After that, the benzene ring of catechol is cleaved by catA via a ring opening reaction and converted to cis, cis-muconate, where the metabolic pathway then leads into the β-KAP. The benzoic acid degradation pathway of strain SP-35 is completely different from that of fungi, which is usually converted to p-hydroxybenzoic acid via benozate 4-monooxygenase and then converted to protocatechuic acid, such as in *Rhodotorula graminis* and *Aspergillus niger* [24]. Interestingly, there are three *benE* and two *benK* genes encoding proteins belonging to the benzoate transporter super family. It was proposed that the products of the *benE* and *benK* genes function as transporters, taking up benzoate into cells to promote the degradation of benzoate [25]. Another interesting finding was that there is a benzoate anaerobic degradation regulator encoding gene (*BadR*) that is able to improve the anaerobic degradation of benzoate [26]. It was suggested that besides the above mentioned benzoate degradation pathway, there may be other anaerobic degradation pathways in strain SP-35. However, strain SP-35 as an aerobic bacterium cannot grow under anerobic conditions using benzoic acid as a single carbon source. Furthermore, the encoding genes of enzymes involving in anaerobic degradation of benzoate such as benzoate-CoA ligase, benzoyl-CoA oxygenase and reductase, benzoyl-CoA hydratease and 3,4-dehydroadipyl-CoA semialdehyde dehydrogenase have not been found in the genome of strain SP-35 [27, 28]. Therefore, the strain SP-35 does not have an anaerobic degradation pathway of benzoate.

3-MP is a precursor to numerous compounds and its metabolic pathway in strain SP-35 was predicted as below. First, 3-MP was converted to 4-methylcatechol via phenol 2-monooxygenase, and then 4-methylcatechol was catalyzed into 3-methyl-cis, cis-muconate by the ring-opening reaction of catA. Previous research showed that catA not only uses catechol as substrate, but also is able to catalyze the methyl-substituted catechols, such as 3-methylcatechol and 4-methylcatechol [29]. However, the phenol 2-monooxygenase gene has not been identified in the genome of strain SP-35 yet and further effort is required to obtain the encode gene of phenol 2-monooxygenase in the future.

Contrary to benzoate and 3-MP, the metabolic pathway of PA was not via β-KAP in the strain SP-35. PA was reduced to phenylacetyl-CoA (PA-CoA) by the reduction catalytic of PA-CoA ligase (*paaK*), and PA-CoA was converted to 1, 2-epoxyphenylacetyl-CoA (ep-CoA) by ring 1,2- phenylacetyl-CoA epoxidase (*paaABCDE*), then ep-CoA was converted to 2-oxepin-2(3H)-ylideneacetyl-CoA by ring 1,2-epoxyphenylacetyl-CoA isomerase (*paaG*). 2-oxepin-2(3H)-ylideneacetyl-CoA was subsequently converted into 3-oxo-5,6-dehydrosuberyl-CoA, which was catalyzed by oxepin-CoA hydrolase (*paaZ*). 3-oxo-5,6-dehydrosuberyl-CoA was converted to 2,3-dehydroadipyl-CoA via the catalyzation of 3-oxoadipyl-CoA thiolase (*paaJ*), then 2,3-dehydroadipyl-CoA was hydrolyzed into 3-hydroxyadipyl-CoA by 2,3-dehydroadipyl-CoA hydratase (*paaF*). The latter intermediate was converted to 3-oxoadipyl-CoA by 3-hydroxyadipyl-CoA dehydrogenase (*paaH*) and finally biotransformed to succinyl-CoA by paaJ. There is one paaK, and five paaG, one paaJ and one paaH encoding genes that were identified in the genome of strain SP-35, which may be involved in the PA degradation pathway. The PA utilization pathway represents one of the strategies for the degradation of a variety of aromatic compounds in bacteria and is different with another strategy which employs two multicomponent monooxygenases or a dioxygenase convert aromatic compounds to catechol and subsequently cleaving the bond and into β-KAP pathway [30]. In addition, unlike the bacterial degradation PA pathway of strain SP-35, the aerobic metabolism of PA by fungi usually catalyzed by monooxygenases and generated homogentisate [31].

As the second major metabolite of MWL, HBA probably was biotransformed from three intermediate metabolites in strain SP-35, including HCA, HBAld and 2, 4′-dihydroxyacetophenone (DHAP). We gathered the pathway from HCA to HBA as follows first, 4-coumarate-CoA ligase catalyzes the ligation of CoA to HCA and produces 4-coumaroyl-CoA, then converts it to 4-hydroxybenzoyl-CoA (HBA-CoA) by beta-oxidation reaction, the beta-oxidation was a multi-step reaction and the enzymes involved in the reaction are still not clear [32], Finally, HBA-CoA was further converted to HBA via HBA-CoA thioesterase. In addition, the HBA was also yielded by the catalysis of HBAld by aldehyde dehydrogenase (ALDH) or from DHAP by DHAP dioxygenase. It is worthwhile mentioning that although most dioxygenases cleave within the aromatic ring of the substrate, DHAP dioxygenase is very unusual in that it is involved in the aliphatic C–C bond cleavage in a substituent of the aromatic ring [33]. The fate of HBA includes two branches, one of which has been converted to 4-hydroxy-3-polyprenylbenzoate by HBA polyprenyltransferase and into ubiquinone biosynthesis pathway via multistep reaction. Another branch of the metabolic pathway is the conversion of HBA to protocatechuate by *p-*hydroxybenzoate hydroxylase, which is then metabolized via the β-KAP.

HAP is the main metabolite of lignin with the highest content in AL culture and its hypothesized metabolic pathway, including a 3-step reaction, is described. The first step is the conversion of HAP to 4-hydroxyphenylacetate (HPA) by the catalysis of 4-hydroxyacetophenone monooxygenase (HAPMO), and then, the catalysis of HPA by *p-*hydroxyphenylacetate 3-hydroxylase, producing 3,4-dihydroxyphenylacetate (HPCA), followed by the ring-opening reaction of catA to convert HPCA to 2-hydroxy-5-carboxymethylmuconate semialdehyde. We found an enzyme encoding gene for Baeyer–Villiger monooxygenase in the genome of SP-35 which might have HAPMO activity. A HAPMO, purified from *Pseudomonas fluorescens* and showing the characteristics of a Baeyer–Villiger-type monooxygenase was reported in the past [34].

The metabolite DHPG has strong antioxidant activity and radical scavenging properties and was found to originate from plants such as the olive fruit as well as medicinal plants [35]. So far there are no published reports on the bacterial depolymerization of lignin to produce DHPG and no information on the bacterial metabolic pathway of DHPG. However, DHPG is an important metabolite in mammalian metabolism, and several metabolic pathways of DHPG were identified in mammalian [36]. Therefore, based on the metabolic pathways of mammals, we surmised the metabolic pathways of DHPG and DOMA as follows. First, the 3,4-dihydroxyphenylglycolaldehyde is converted to DHPG or DOMA by the catalysis of alcohol dehydrogenase (ADH) or ALDH, respectively. Then the DHPG is converted to 3-methoxy-4-hydroxyphenylglycol (MHPG) via catechol *O*-methyltransferase (COMT). After that, MHPG is converted to 3-methoxy-4-hydroxyphenylglycolaldehyde (MOPEGAL) by ADH and is then biotransformed to 3-methoxy-4-hydroxymandelate (VMA) by ALDH. DOMA was also converted to VMA via COMT. The COMT has already been identified in bacteria, which have similar substrates with those of mammals [37]. Even though we have found six ALDH and nine ADH encoding genes in the genome of strain SP-35, their catalytic function in the metabolic pathways of DHPG and DOMA remains to be further confirmed.

Ferulic acid is an extremely abundant phenolic cinnamic acid derivative in the plant kingdom, and ferulic acid and ρ-coumaric acids are predominantly linked at the benzyl position of lignin. As shown in Fig. 8, the hypothetical degradation pathway of ferulic acid in the strain SP-35 is discussed below. Ferulic acid is biotransformed into vanillic acid with feruloyl-CoA and vanillin as intermediates, by the catalytic functions of feruloyl-CoA synthetase and vanillin dehydrogenase, respectively. The metabolism of vanillic acid is described in the conversion of *O*-demethylated to protocatechuate. Another possible pathway for the degradation of vanillic acid is the conversion of vanillic acid to guaiacol by vanillate decarboxylase followed by the biotransformation of guaiacol to catechol by the aromatic ring-*O*-demethylase [38, 39]. Interestingly, experiments of a single-carbon source growth of strain SP-35 showed that when 0.5 g/L aromatic compound was added as a carbon source, the ferulic acid, vanillin, vanillic acid and protocatechuate were exhausted at 48, 72, 36, and 24 h, respectively (Table 3). The fact that the conversion of vanillin to vanillic acid needed a longer time suggests that this is the rate-limiting step in the ferulic acid decomposition pathway. In addition, there is another biosynthesis pathway for vanillin in bacteria that is obtained by the conversion of ρ-coumaric acid via two intermediates [40]. However, there were no identified enzymes involved in the ρ-coumaric acid pathway in the genome of strain SP-35, and ρ-coumaric acid cannot be effectively used as the sole carbon source to support cell growth (Table 3). Our results indicate that the vanillin was mainly obtained from the ferulic acid pathway and the β-aryl ether pathways in the strain SP-35.

SA is the only metabolite identified in the MWL culture that belongs to the S type. The proposed pathway for the metabolism of SA starts with the conversion of SA to syringate by ALDH and the subsequent conversion of syringate to 3-*O*-methylgallate (3MGA) by vanillate/syringate *O*-demethylase (V/SODM). The compound 3MGA was able to be biotransformed to gallate by V/SODM. Four ALDH genes were observed in the genome of strain SP-35, among them, one is involved in the degradation of SA, which needs to be verified in the future. The degradation of syringate in *Sphingomonas paucimobilis* SYK-6 involves the participation of two V/SODM, LigM and desA. The substrate of LigM is 3MGA and the substrate of desA is syringate [41]. Correspondingly, there were also two V/SODM-encoding genes in strain SP-35 that were identified, which may have encoded the enzyme that degrades syringate and the enzyme that degrades 3MGA, respectively. In addition, 3MGA was able to be degraded through multiple pathways and was able to be converted into different products, including 2-pyrone-4,6-dicarboxylate and 4-carboxy-2-hydroxy-6-methoxy-6-oxohexa-2,4-dienoate via protocatechuate 4, 5-dioxygenase and 3MGA 3, 4-dioxygenase [42, 43]. However, the genes encoding protocatechuate 4, 5-dioxygenase or 3MGA 3, 4-dioxygenase still have not been identified in strain SP-35. Notably, ALDH has a wide range of substrates and is able to degrade benzaldehyde and its derivatives, such as SA, vanillin, benzaldehyde, *p*-hydroxybenzaldehyde, protocatechualdehyde, *m*-anisaldehyde, veratraldehyde, coniferyl aldehyde, salicylaldehyde, *m*-hydroxybenzaldehyde, etc. [44]. Therefore, the extensiveness of the enzyme’s possible substrates has led to difficulties in elucidating the metabolic pathways of lignin-derived aromatic compounds, and the diversity of lignin degradation intermediates has also increased the complexity of the lignin metabolism network in bacteria.

Enzymes involved in lignin degradation are quite different between strain SP-35 and fungi. For example, lignin-modifying enzymes produced by fungus including Lacc, LiP, MnP, VP and DyPs, while the strain SP-35 only include Lacc and DyPs. Lignin-degrading auxiliary enzymes produced by fungus including glyoxal oxidase, aryl alcohol oxidases, pyranose 2-oxidase, cellobiose dehydrogenase and glucose oxidase, but all above enzymes in the strain SP-35 were not found [45]. Usually, hydrogen peroxide was necessary for lignin degradation by LiP, MnP and VP of fungus, but DyP from bacteria was proved able to degrade lignin without hydrogen peroxide [46].

## Conclusions

In this report, we described a detailed study of the degradation of MWL and AL by the strain SP-35, which could be an efficient cell factory for the conversion of lignin to high-value aromatic compounds. In summary, there are at least five enzymes in strain SP-35 involved in the oxidation and depolymerization of lignin including DyPs, and Lacc, etc. The variations of absorption of FT-IR spectra indicate that the degradation of MWL by strain SP-35 included the oxidative cleavage of the lignin aromatic ring structures and the modification of its side chains. Based on genome analysis and the intermediate metabolites, we speculated multiple aromatic compounds’ metabolic pathways in strain SP-35, including benzoate, phenylacetic acid, 4-hydroxycinnamic acid, 4-hydroxyacetophenone, 3, 4-dihydroxyphenylglycol, ferulic acid, and syringaldehyde, and a DPHG degradation pathway first identified in bacteria, etc. In addition, the β-aryl ether cleavage pathway was first described in bacteria other than the family Sphingomonadaceae. A total of 94 genes were identified to encode 34 enzymes and 5 regulatory factors involved in lignin degradation and catabolism of its intermediates. It was suggested that the degradation of lignin constitutes a complex metabolic network in strain SP-35.

The large-scale production of value-added aromatic compounds released by SP-35, such as vanillin, SA, coumaric acid, catechol and protocatechuic acid is fascinating if the bottleneck of genetic manipulation was tackled. Therefore, the future work will focus on inactivation of vanillin dehydrogenase, aldehyde dehydrogenase, CatA and PcaA enzyme activities by gene knockout gene editing or other synthetic biology methods, thereby blocking the metabolic pathway of vanillin, SA, coumaric acid, β-KAP achieves the purpose of accumulating high-value aromatic compounds on large-scale.

## Additional files

**Additional file 1.** The tolerance of strain SP-35 cultrue for aromatic compounds.

**Additional file 2.** Genome annotation of *Comamonas serinivorans* SP-35.

## Abbreviations

MWL: milled wood lignin
AL: alkali lignin
H: ρ-hydroxyphenyl
G: guaiacyl
S: syringyl
LiP: lignin peroxidases
MnP: manganese peroxidases
VP: versatile peroxidases
DyPs: dye decolorizing peroxidases
Lacc: laccases
β-KAP: β-ketoadipate pathway
LB: Luria broth
LM: lignin medium
COD: chemical oxygen demand
SEM: scanning electron microscopy
FTIR: Fourier-transform infrared spectrometer
GC–MS: gas chromatography–mass spectrometry
BSTFA: *N*, *O*-bis (trimethylsilyl) trifluoroacetamide
TMCS: trimethylchlorosilane
TMS: trimethylsilyl
SMRT: single molecule real time
HCA: 4-hydroxycinnamic acid
2,4-DHB: 2,4-dihydroxybenzaldehyde
HAP: 4′-hydroxyacetophenone
DHPG: 3,4-dihydroxyphenylglycol
HBA: 4-hydroxybenzoic acid
DOMA: 3,4-dihydroxymandelic acid
3-MP: 3-methylphenol
BHT: butylated hydroxytoluene
GSTs: glutathione transferases
NAD-ADH: NAD-dependent alcohol dehydrogenase
GGE: guaiacylglycerol-β-guaiacyl ether
MPHPV: α-(2-methoxyphenoxy)-β-hydroxypropiovanillone
GS-HPV: generateβ-glutathionyl-γ-hydroxypropiovanillone
GSSG: glutathione disulfide
HPV: γ-hydroxypropiovanillone
VVA: vanilloyl acetic acid
PA: phenylacetic acid
HBAld: 4-hydroxybenzaldehyde
SA: syringaldehyde
catA: catechol 1, 2-dioxygenase
catB: muconate cycloisomerase
catC: muconolactone D-isomerase
pcaD: β-ketoadipate enol-lactone hydrolase
pcaI: 3-oxoadipate CoA-transferase subunit A
pcaJ: 3-oxoadipate CoA-transferase subunit B
pcaF: β-ketoadipyl CoA thiolase
pcaC: carboxymuconolactone decarboxylase
pcaHG: protocatechuate 3, 4-dioxygenase
pcaB: β-carboxymuconate cycloisomerase
pcaR: β-KAP transcription regulator
DHCDC: cis-1, 2-dihydroxycyclohexa-3, 5-diene-1-carboxylate
BadR: benzoate anaerobic degradation regulator
PA-CoA: phenylacetyl-CoA
ep-CoA: 1, 2-epoxyphenylacetyl-CoA
DHAP: 2, 4′-dihydroxyacetophenone
HBA-CoA: 4-hydroxybenzoyl-CoA
ALDH: aldehyde dehydrogenase
HPA: 4-hydroxyphenylacetate
HAPMO: 4-hydroxyacetophenone monooxygenase
HPCA: 3,4-dihydroxyphenylacetate
ADH: alcohol dehydrogenase
MHPG: 3-methoxy-4-hydroxyphenylglycol
COMT: catechol *O-*methyltransferase
MOPEGAL: 3-methoxy-4-hydroxyphenylglycolaldehyde
VMA: 3-methoxy-4-hydroxymandelate
3MGA: 3-*O*-methylgallate
V/SODM: vanillate/syringate *O*-demethylase
