# Supplementary material for: Genomics and biochemistry investigation on the metabolic pathway of milled wood and alkali lignin-derived aromatic metabolites of Comamonas serinivorans SP-35
Source: Biotechnol Biofuels. 2018 Dec 27;11:338. doi: 10.1186/s13068-018-1341-3 (PMC6307125; doi:10.1186/s13068-018-1341-3)
Supplement: Supplementary file 1 — Additional file 1. The tolerance of strain SP-35 cultrue for aromatic compounds. [file 13068_2018_1341_MOESM1_ESM.docx]

| Table S1. The tolerance of strain SP-35 cultrue for aromatic compounds* | | | | |
| --- | --- | --- | --- | --- |
| Compound | tolerant concentration(g/L) | | | |
|  | 0.1 | 0.5 | 1 | 5 |
| guaiacol | + | + | - |  |
| 3-methylphenol | + | - |  |  |
| 3-o-methylgallate | + | + | + | - |
| 4-hydroxybenzaldehyde | + | + | - |  |
| 2,4-dihydroxybenzaldehyde | + | - |  |  |
| catechol | + | + | + | - |
| butylated hydroxytoluene | + | + | + |  |
| methyl 2,5-dihydroxybenzoate | + | + | - |  |
| 4-hydroxybenzoate | + | - |  |  |
| vanillin | + | + | - |  |
| acetovanillone | + | + | - |  |
| 4'-hydroxyacetophenone | + | - |  |  |
| vanillic acid | + | + | + | - |
| 3,4-dihydroxymandelic acid | + | + | - |  |
| syringaldehyde | + | + | + | - |
| protocatechuate | + | + | - |  |
| dihydroferulic acid | + | + | - |  |
| 4-hydroxycinnamic acid | + | - |  |  |
| benzoate | + | + | - |  |
| ferulic acid | + | - |  |  |
| *cells were incuabated in LB medium with different aromatic compounds at 30℃ for 7 days,"+", cells are grow; “-”， cells were not grow. | | | | |
